# University of Ottawa constant and variable speed electric motor vibration and acoustic fault signature dataset

**DOI:** 10.1016/j.dib.2024.110144

**Published:** 2024-02-02

**Authors:** Mert Sehri, Patrick Dumond

**Affiliations:** Department of Mechanical Engineering, University of Ottawa, 161 Louis Pasteur, Ottawa, Ontario, Canada

**Keywords:** Induction motor, Electrical faults, Mechanical faults, Vibration, Acoustic, Condition monitoring, Signal processing

## Abstract

Induction motors are used in industry as they are self-starting, reliable, and affordable. Applications for these motors include lathes, mills, pumps, power conveyor belts, and commercial electrical and hybrid vehicles. Induction motors have various types of failures, including rotor unbalance, rotor misalignment, stator winding faults, voltage unbalance, bowed rotor, broken rotor bars, and faulty bearings. There is a need for differentiating mechanical faults from electrical fault signals when identifying what part of the motor needs maintenance while using machine learning. Therefore, data collection is essential for electric motor fault diagnosis. The University of Ottawa Electric Motor Dataset – Vibration and Acoustic Faults under Constant and Variable Speed Conditions (UOEMD-VAFCVS) is provided to address this issue. Data from accelerometers, temperature, and acoustic sensors are collected to provide quality electric motor fault data. The dataset includes various induction motor faults useful for time domain analysis. The high-quality data provided by this dataset will help facilitate the differentiation between mechanical faults and electric faults when using fault detection methods, which is a valuable asset for machine condition monitoring.

Specifications Table

**Table:** 

Subject	Electrical and Mechanical Engineering
Specific subject area	Fault detection of rotor unbalance, rotor misalignment, stator winding faults, voltage unbalance, bowed rotor, broken rotor bars, and faulty bearings. Time series vibration data (single-axis)
Data Format	Raw
Type of data	.*csv* (dataset with numbers) .mat (dataset with numbers)
Data Collection	Data was acquired from a modified version of the SpectraQuest Machinery Fault & Rotor Dynamics Simulator test rig. Three accelerometers, a microphone, and a variable frequency drive were attached to the test rig to collect vibration, acoustic, temperature, and rotational speed data using LabVIEW. This dataset includes a healthy motor, rotor unbalance, rotor misalignment, stator winding fault, voltage unbalance and single phasing, bowed rotor, broken rotor bars, and faulty bearings. In addition, electric motor health condition data under constant and variable speeds are provided. 420,000 samples are collected at a sampling rate of 42,000 Hz.
Data source location	Institution: University of Ottawa City/Town/Region: Ottawa, Ontario Country: Canada
Data accessibility	**Repository name:** Mendeley Data **Data identification number:** ***Raw Data doi:***10.17632/msxs4vj48g.1 **Direct URL to data:** Raw Data: https://data.mendeley.com/datasets/msxs4vj48g

## 1 Value of the Data

• The time domain signal obtained from the data can be applied with different fault signal methodologies, like spectral analysis or time-frequency analysis, to distinguish between various mechanical and electrical induction motor fault types.
• The data can be useful for exploring the correlation between variable speed and constant speed datasets when considering different fault identification methodologies. This exploration can provide insights into how faults differ under different operating conditions.
• The data collected can be used to train machine learning algorithms for electric motor fault diagnosis, either in the raw format or after being processed. This training can involve algorithms like neural networks and support vector machines.
• The data can be used for phase analysis due to accelerometer placements when determining electrical faults. Phase analysis involves understanding how accelerometer placements contribute to accurate fault analysis.
• The data provides noisy and clean signals to encourage fault detection and extraction under both regimes. Noisy signals challenge the detection process, while clean signals act as a reference for comparison, ensuring effective fault extraction.
• The electrical dataset provided in this article can be combined with other mechanical fault datasets, such as [1], [2], [3], [4], to differentiate between electrical and mechanical faults.

## 2 Background

An induction motor test rig operating at constant and variable speeds collects signals from accelerometers, a temperature sensor, a microphone, and a variable frequency drive. The dataset provided herein encourages researchers to use traditional and deep learning approaches to identify mechanical and electrical faults in induction motors. The data will enable the application of various methodologies and algorithms to diagnose electrical and mechanical motor issues. The data will aid in the training and validating of deep learning algorithms, hence improving the accuracy of machine learning methods and encouraging the usage of different fault type identification methods [5,6]. The dataset uses different conditions, including unloaded, loaded, constant, and variable speeds.

## 3 Data Description

The individual files contain 10 s of data each at a sampling rate of 42,000 Hz. In each file, the first column provides data from the accelerometer on the drive end of the motor, the second column is acoustic data, the third column provides data from the second accelerometer on the shaft's bearing housing near the drive end of the motor, the fourth column provides data for the third accelerometer on the shaft's bearing housing furthest from the drive end of the motor, and the fifth column is the temperature data from the motor surface at the drive end. The raw data is provided as time series vibration, acoustic, and temperature amplitudes, whereas motor speeds are provided as rotational frequencies. The format of the set of data is as follows: {Letter}-{Letter}-{Number}-{Number}.

The first and second letters in the dataset labeling scheme are combined to indicate the health condition of the electric motor being tested. Specifically, the first letter can be denoted as “H” for healthy, “R” for rotor, “S” for stator, “V” for voltage, “B” for bowed, “K” for broken, or “F” for faulty. The second letter in the dataset labeling scheme can be denoted as “H” for healthy, “U” for unbalance, “M” for misalignment, “W” for winding, “R” for rotor, “A” for rotor bars, or “B” for bearing.

The first number in the dataset labeling scheme identifies the operating frequency of the electric motor. On the other hand, the last number in the dataset labeling scheme represents whether the motor is unloaded or loaded, where “0” represents an unloaded motor shaft, and “1” represents a loaded motor shaft.

For example, the data sample labeled “R-U-1–0” corresponds to an unloaded rotor unbalance fault at a motor speed of 15 Hz, whereas “R-M-4-1” represents a loaded rotor misalignment fault at 60 Hz. Similarly, “B-R-7-0” represents an unloaded bowed rotor fault at speeds decreasing from 45 Hz to 15 Hz, while “V-U-5-1” indicates a loaded voltage unbalance at speeds increasing from 15 Hz to 45 Hz.

Tables 1 and 2 provide a rundown of the dataset's labeling.

**Table 1:** Dataset labeling for constant speed conditions.

Motor load condition	Motor health condition	Constant Speed			
		15 Hz	30 Hz	45 Hz	60 Hz
Unloaded	Healthy	H-H-1-0	H-H-2-0	H-H-3-0	H-H-4-0
	Rotor Unbalance	R-U-1-0	R-U-2-0	R-U-3-0	R-U-4-0
	Rotor Misalignment	R-M-1-0	R-M-2-0	R-M-3-0	R-M-4-0
	Stator Winding Fault	S-W-1-0	S-W-2-0	S-W-3-0	S-W-4-0
	Voltage Unbalance and Single Phasing	V-U-1-0	V-U-2-0	V-U-3-0	V-U-4-0
	Bowed Rotor	B-R-1-0	B-R-2-0	B-R-3-0	B-R-4-0
	Broken Rotor Bars	K-A-1-0	K-A-2-0	K-A-3-0	K-A-4-0
	Faulty Bearings	F-B-1-0	F-B-2-0	F-B-3-0	F-B-4-0
Loaded	Healthy	H-H-1-1	H-H-2-1	H-H-3-1	H-H-4-1
	Rotor Unbalance	R-U-1-1	R-U-2-1	R-U-3-1	R-U-4-1
	Rotor Misalignment	R-M-1-1	R-M-2-1	R-M-3-1	R-M-4-1
	Stator Winding Fault	S-W-1-1	S-W-2-1	S-W-3-1	S-W-4-1
	Voltage Unbalance and Single Phasing	V-U-1-1	V-U-2-1	V-U-3-1	V-U-4-1
	Bowed Rotor	B-R-1-1	B-R-2-1	B-R-3-1	B-R-4-1
	Broken Rotor Bars	K-A-1-1	K-A-2-1	K-A-3-1	K-A-4-1
	Faulty Bearings	F-B-1-1	F-B-2-1	F-B-3-1	F-B-4-1

**Table 2:** Dataset labeling for variable speed conditions.

Motor load condition	Motor health condition	Variable Speed			
		15 to 45 Hz	30 to 60 Hz	45 Hz to 15 Hz	60 Hz to 30 Hz
Unloaded	Healthy	H-H-5-0	H-H-6-0	H-H-7-0	H-H-8-0
	Rotor Unbalance	R-U-5-0	R-U-6-0	R-U-7-0	R-U-8-0
	Rotor Misalignment	R-M-5-0	R-M-6-0	R-M-7-0	R-M-8-0
	Stator Winding Fault	S-W-5-0	S-W-6-0	S-W-7-0	S-W-8-0
	Voltage Unbalance and Single Phasing	V-U-5-0	V-U-6-0	V-U-7-0	V-U-8-0
	Bowed Rotor	B-R-5-0	B-R-6-0	B-R-7-0	B-R-8-0
	Broken Rotor Bars	K-A-5-0	K-A-6-0	K-A-7-0	K-A-8-0
	Faulty Bearings	F-B-5-0	F-B-6-0	F-B-7-0	F-B-8-0
Loaded	Healthy	H-H-5-1	H-H-6-1	H-H-7-1	H-H-8-1
	Rotor Unbalance	R-U-5-1	R-U-6-1	R-U-7-1	R-U-8-1
	Rotor Misalignment	R-M-5-1	R-M-6-1	R-M-7-1	R-M-8-1
	Stator Winding Fault	S-W-5-1	S-W-6-1	S-W-7-1	S-W-8-1
	Voltage Unbalance and Single Phasing	V-U-5-1	V-U-6-1	V-U-7-1	V-U-8-1
	Bowed Rotor	B-R-5-1	B-R-6-1	B-R-7-1	B-R-8-1
	Broken Rotor Bars	K-A-5-1	K-A-6-1	K-A-7-1	K-A-8-1
	Faulty Bearings	F-B-5-1	F-B-6-1	F-B-7-1	F-B-8-1

**Fig. 1:**
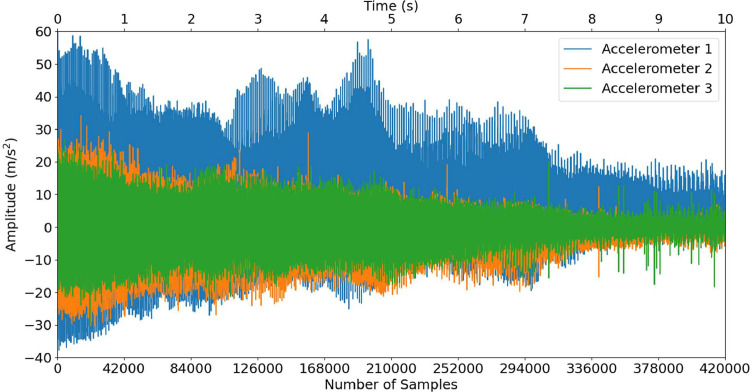
Accelerometer data for B-R-7-0 (Decreasing Speed).

**Fig. 2:**
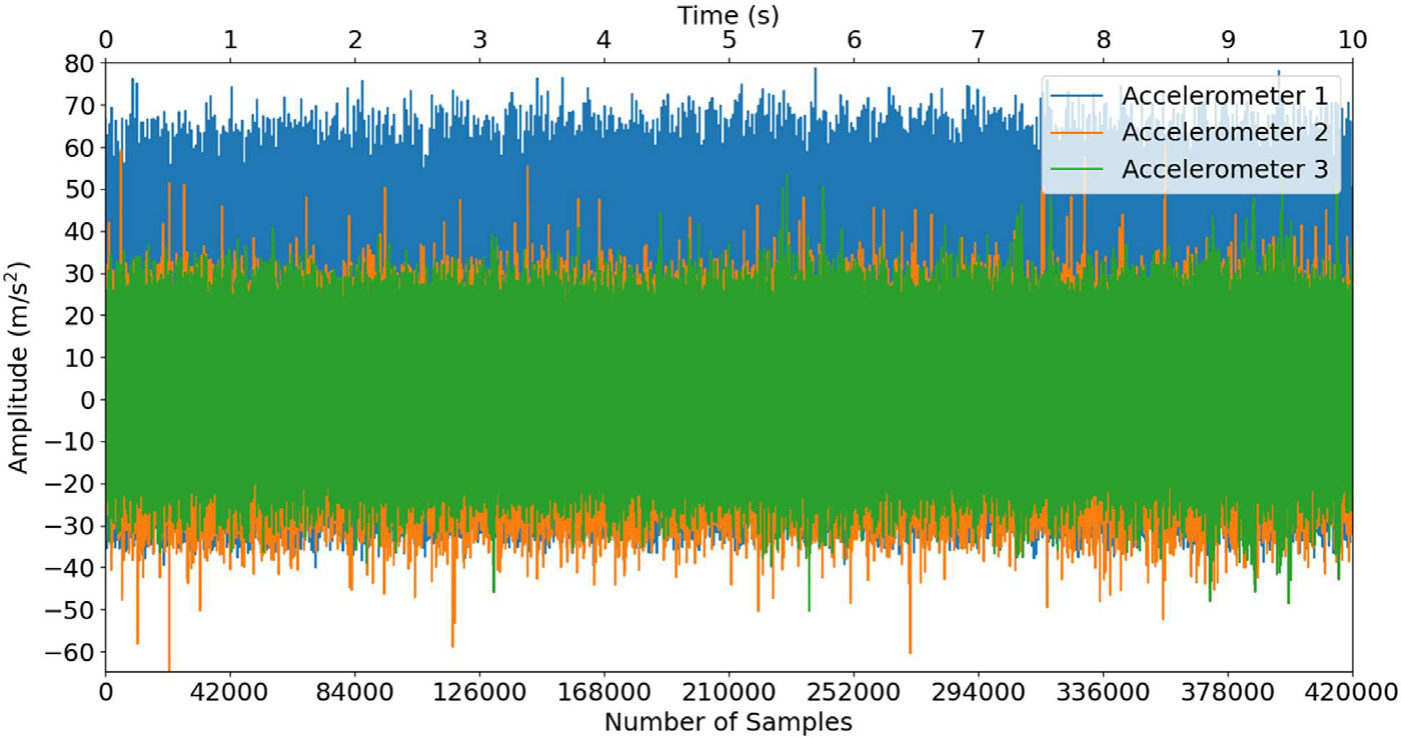
Accelerometer data for B-R-4-0 (Constant Speed).

A time series sample of B-R-7-0 (broken rotor bar from 45 Hz to 15 Hz) is shown in Fig. 1 (vibration data), and a sample of B-R-4-0 (broken rotor bar at 60 Hz) is shown in Fig. 2 (vibration data).

The data for the healthy electric motor, as well as for the different electric motor fault types, were recorded using approximately constant frequencies (∼15 Hz, ∼30 Hz, ∼45 Hz, and ∼60 Hz) and variable frequencies (∼15 Hz to ∼45 Hz, ∼30 Hz to ∼60 Hz, ∼45 to ∼15 Hz, and ∼60 Hz to ∼30 Hz). A detailed breakdown of the dataset is provided below.

No load conditions:

­ H-H-1-0: operating frequency of 15.10 Hz
­ H-H-2-0: operating frequency of 29.98 Hz
­ H-H-3-0: operating frequency of 44.91 Hz
­ H-H-4-0: operating frequency of 59.80 Hz
­ H-H-5-0: operating frequency increased from 14.99 Hz to 44.86 Hz
­ H-H-6-0: operating frequency increased from 29.83 Hz to 59.80 Hz
­ H-H-7-0: operating frequency decreased from 44.89 Hz to 15.12 Hz
­ H-H-8-0: operating frequency decreased from 59.79 Hz to 30.57 Hz
­ R-U-1-0: operating frequency of 15.11 Hz
­ R-U-2-0: operating frequency of 30.03 Hz
­ R-U-3-0: operating frequency of 45.67 Hz
­ R-U-4-0: operating frequency of 59.95 Hz
­ R-U-5-0: operating frequency increased from 15.72 Hz to 44.97 Hz
­ R-U-6-0: operating frequency increased from 30.34 Hz to 59.88 Hz
­ R-U-7-0: operating frequency decreased from 44.92 Hz to 16.11 Hz
­ R-U-8-0: operating frequency decreased from 59.98 Hz to 30.72 Hz
­ R-M-1-0: operating frequency of 15.16 Hz
­ R-M-2-0: operating frequency of 29.67 Hz
­ R-M-3-0: operating frequency of 44.92 Hz
­ R-M-4-0: operating frequency of 59.91 Hz
­ R-M-5-0: operating frequency increased from 15.82 Hz to 44.91 Hz
­ R-M-6-0: operating frequency increased from 30.33 Hz to 59.96 Hz
­ R-M-7-0: operating frequency decreased from 44.97 Hz to 18.82 Hz
­ R-M-8-0: operating frequency decreased from 59.95 Hz to 30.22 Hz
­ S-W-1-0: operating frequency of 15.08 Hz
­ S-W-2-0: operating frequency of 30.27 Hz
­ S-W-3-0: operating frequency of 45.04 Hz
­ S-W-4-0: operating frequency of 59.91 Hz
­ S-W-5-0: operating frequency increased from 15.41 Hz to 44.96 Hz
­ S-W-6-0: operating frequency increased from 29.78 Hz to 59.88 Hz
­ S-W-7-0: operating frequency decreased from 44.92 Hz to 15.65 Hz
­ S-W-8-0: operating frequency decreased from 59.91 Hz to 30.23 Hz
­ V-U-1-0: operating frequency of 14.92 Hz
­ V-U-2-0: operating frequency of 30.17 Hz
­ V-U-3-0: operating frequency of 44.96 Hz
­ V-U-4-0: operating frequency of 59.91 Hz
­ V-U-5-0: operating frequency increased from 15.14 Hz to 44.96 Hz
­ V-U-6-0: operating frequency increased from 30.08 Hz to 59.88 Hz
­ V-U-7-0: operating frequency decreased from 44.91 Hz to 15.32 Hz
­ V-U-8-0: operating frequency decreased from 59.90 Hz to 29.82 Hz
­ B-R-1-0: operating frequency of 15.22 Hz
­ B-R-2-0: operating frequency of 30.17 Hz
­ B-R-3-0: operating frequency of 44.93 Hz
­ B-R-4-0: operating frequency of 59.94 Hz
­ B-R-5-0: operating frequency increased from 15.22 Hz to 44.87 Hz
­ B-R-6-0: operating frequency increased from 29.88 Hz to 59.77 Hz
­ B-R-7-0: operating frequency decreased from 44.92 Hz to 15.75 Hz
­ B-R-8-0: operating frequency decreased from 59.90 Hz to 30.36 Hz
­ K-A-1-0: operating frequency of 15.41 Hz
­ K-A-2-0: operating frequency of 30.45 Hz
­ K-A-3-0: operating frequency of 44.77 Hz
­ K-A-4-0: operating frequency of 59.89 Hz
­ K-A-5-0: operating frequency increased from 15.13 Hz to 44.87 Hz
­ K-A-6-0: operating frequency increased from 29.66 Hz to 59.94 Hz
­ K-A-7-0: operating frequency decreased from 44.96 Hz to 16.81 Hz
­ K-A-8-0: operating frequency decreased from 59.97 Hz to 30.21 Hz
­ F-B-1-0: operating frequency of 15.06 Hz
­ F-B-2-0: operating frequency of 30.93 Hz
­ F-B-3-0: operating frequency of 44.91 Hz
­ F-B-4-0: operating frequency of 59.90 Hz
­ F-B-5-0: operating frequency increased from 15.22 Hz to 44.96 Hz
­ F-B-6-0: operating frequency increased from 30.71 Hz to 59.88 Hz
­ F-B-7-0: operating frequency decreased from 44.90 Hz to 14.44 Hz
­ F-B-8-0: operating frequency decreased from 59.95 Hz to 28.82 Hz

Loaded conditions:

­ H-H-1-1: operating frequency of 15.52 Hz
­ H-H-2-1: operating frequency of 30.87 Hz
­ H-H-3-1: operating frequency of 44.86 Hz
­ H-H-4-1: operating frequency of 59.95 Hz
­ H-H-5-1: operating frequency increased from 15.11 Hz to 44.91 Hz
­ H-H-6-1: operating frequency increased from 30.69 Hz to 59.98 Hz
­ H-H-7-1: operating frequency decreased from 44.89 Hz to 14.52 Hz
­ H-H-8-1: operating frequency decreased from 59.95 Hz to 29.18 Hz
­ R-U-1-1: operating frequency of 15.32 Hz
­ R-U-2-1: operating frequency of 30.27 Hz
­ R-U-3-1: operating frequency of 44.91 Hz
­ R-U-4-1: operating frequency of 59.97 Hz
­ R-U-5-1: operating frequency increased from 15.88 Hz to 44.99 Hz
­ R-U-6-1: operating frequency increased from 30.61 Hz to 59.93 Hz
­ R-U-7-1: operating frequency decreased from 44.97 Hz to 16.13 Hz
­ R-U-8-1: operating frequency decreased from 59.91 Hz to 29.11 Hz
­ R-M-1-1: operating frequency of 15.41 Hz
­ R-M-2-1: operating frequency of 30.43 Hz
­ R-M-3-1: operating frequency of 45.48 Hz
­ R-M-4-1: operating frequency of 59.97 Hz
­ R-M-5-1: operating frequency increased from 15.11 Hz to 44.96 Hz
­ R-M-6-1: operating frequency increased from 30.55 Hz to 59.90 Hz
­ R-M-7-1: operating frequency decreased from 44.95 Hz to 15.33 Hz
­ R-M-8-1: operating frequency decreased from 59.91 Hz to 29.71 Hz
­ S-W-1-1: operating frequency of 15.21 Hz
­ S-W-2-1: operating frequency of 30.17 Hz
­ S-W-3-1: operating frequency of 45.05 Hz
­ S-W-4-1: operating frequency of 59.98 Hz
­ S-W-5-1: operating frequency increased from 15.33 Hz to 44.92 Hz
­ S-W-6-1: operating frequency increased from 30.66 Hz to 59.97 Hz
­ S-W-7-1: operating frequency decreased from 44.91 Hz to 15.88 Hz
­ S-W-8-1: operating frequency decreased from 59.91 Hz to 32.41 Hz
­ V-U-1-1: operating frequency of 15.66 Hz
­ V-U-2-1: operating frequency of 30.71 Hz
­ V-U-3-1: operating frequency of 44.93 Hz
­ V-U-4-1: operating frequency of 59.95 Hz
­ V-U-5-1: operating frequency increased from 15.72 Hz to 44.91 Hz
­ V-U-6-1: operating frequency increased from 30.44 Hz to 59.87 Hz
­ V-U-7-1: operating frequency decreased from 44.98 Hz to 15.11 Hz
­ V-U-8-1: operating frequency decreased from 59.90 Hz to 29.79 Hz
­ B-R-1-1: operating frequency of 15.22 Hz
­ B-R-2-1: operating frequency of 30.37 Hz
­ B-R-3-1: operating frequency of 45.01 Hz
­ B-R-4-1: operating frequency of 59.97 Hz
­ B-R-5-1: operating frequency increased from 15.81 Hz to 44.81 Hz
­ B-R-6-1: operating frequency increased from 29.95 Hz to 59.91 Hz
­ B-R-7-1: operating frequency decreased from 44.86 Hz to 18.32 Hz
­ B-R-8-1: operating frequency decreased from59.93 Hz to 30.71 Hz
­ K-A-1-1: operating frequency of 15.32 Hz
­ K-A-2-1: operating frequency of 30.47 Hz
­ K-A-3-1: operating frequency of 44.95 Hz
­ K-A-4-1: operating frequency of 59.97 Hz
­ K-A-5-1: operating frequency increased from 15.41 Hz to 44.96 Hz
­ K-A-6-1: operating frequency increased from 29.66 Hz to 59.87 Hz
­ K-A-7-1: operating frequency decreased from 44.97 Hz to 14.74 Hz
­ K-A-8-1: operating frequency decreased from 59.94 Hz to 30.02 Hz
­ F-B-1-1: operating frequency of 15.61 Hz
­ F-B-2-1: operating frequency of 30.43 Hz
­ F-B-3-1: operating frequency of 45.22 Hz
­ F-B-4-1: operating frequency of 59.95 Hz
­ F-B-5-1: operating frequency increased from 15.47 Hz to 44.87 Hz
­ F-B-6-1: operating frequency increased from 30.61 Hz to 59.90 Hz
­ F-B-7-1: operating frequency decreased from 44.97 Hz to 15.82 Hz
­ F-B-8-1: operating frequency decreased from 59.95 Hz to 28.51 Hz

The accelerometers and temperature values are captured in V, and the data is then converted to $\frac{m}{s^{2}}\;and{\;}^{\circ }C$ using the sensitivity conversion rates provided by the manufacturer. Therefore, the data presented in each raw data file have the following units: vibration [accelerometer 1] ($\frac{m}{s^{2}}$), acoustic sound (V), vibration [accelerometer 2] ($\frac{m}{s^{2}})$, vibration [accelerometer 3] ($\frac{m}{s^{2}})$, temperature [accelerometer 1] (${}^{\circ }C$).

## 4 Experimental Design, Materials, and Methods

Due to the use of an acoustic sensor, a sampling frequency of 42,000 Hz is selected based on the audible frequency range of 20,000 Hz and by applying the Shannon-Nyquist theorem [7,8]. The data collection time is set to 10 s to have enough samples to distinguish between increasing and decreasing speeds. Therefore, 420,000 data samples are collected for each set of data. Every electric motor tested has its individual artificially created fault (fault included by SpectraQuest), allowing for the collection of different results. The dataset is designed to contain four different motor speeds for each type of fault at both constant and variable operating speeds. A total of 8 electric motors (same model) are used to collect the data (1 healthy and 7 faulty motors). The electric motor data for each set was collected using sensor placements meant to reduce the amount of noise contained within the collected signals at both the drive end and the shaft bearing housings.

The specifications of the motor model used in this dataset are provided in Table 3.

**Table 3:** Motor specifications.

Model	Frequency	Output power	Max Speed	Phases	Electrical type	Motor Bearings
Marathon Electric D396	60.00 Hz	3.00 HP	3600 RPM	3	Induction	6205

**Fig. 3:**
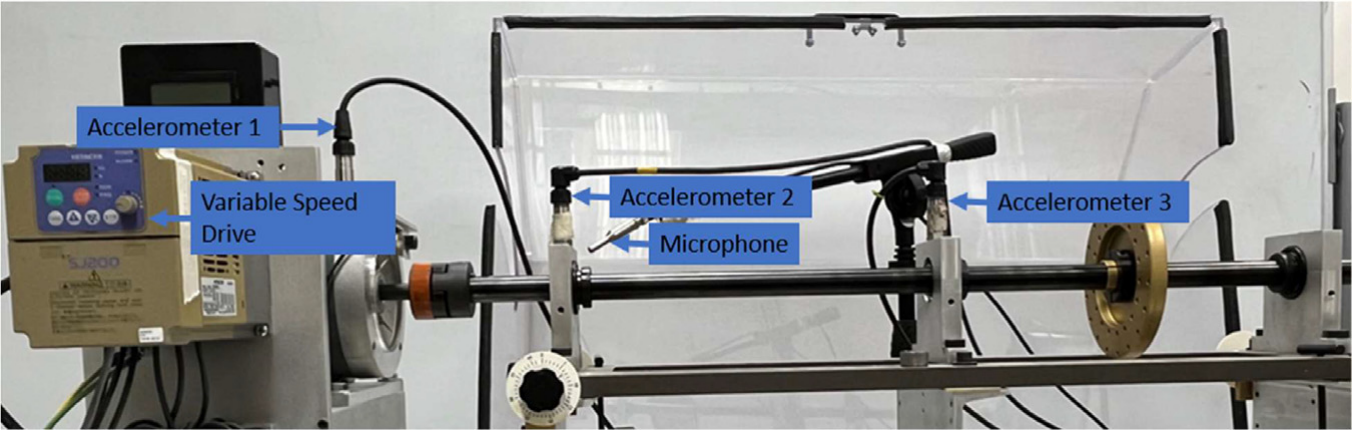
Test rig setup.

A total of 128 sets of data are available at https://data.mendeley.com/datasets/msxs4vj48g [9]. Data is collected using a modified version of the SpectraQuest Machinery Fault & Rotor Dynamics Simulator test rig. The experimental setup is shown in Fig. 3. The experimental setup includes a 3-phase motor mounted on a rigid plate supported by rubber dampers. A variable frequency drive controls the motor's rotational speed, measured in Hz.

The test rig setup in Fig. 3 has four sensors and a variable speed controller: three accelerometers, a microphone, and a variable speed drive. Accelerometer 1 [with an integrated temperature sensor] (PCB, model 603C01) is mounted to the motor drive end using a magnet. Accelerometers 2 and 3 (PCB, model 623C01) are mounted on the shaft's bearing housings on the left and right of Fig. 3, respectively, using stud mounts. The microphone (PCB, model 130F20) is placed within 2 cm of the left bearing housing, it is supported using an independent and vibrationally isolated stand.

This setup allows fault vibration signatures to be collected on both the motor drive end and at two locations along the shaft, providing a reduced signal-to-noise ratio. Accelerometer 2 and 3 placements, shown in Fig. 3, provide researchers with clean electric motor signals with a minimum amount of noise. In contrast, the signal obtained from accelerometer 1 has electrical noise coming from the variable speed drive.

**Fig. 4:**
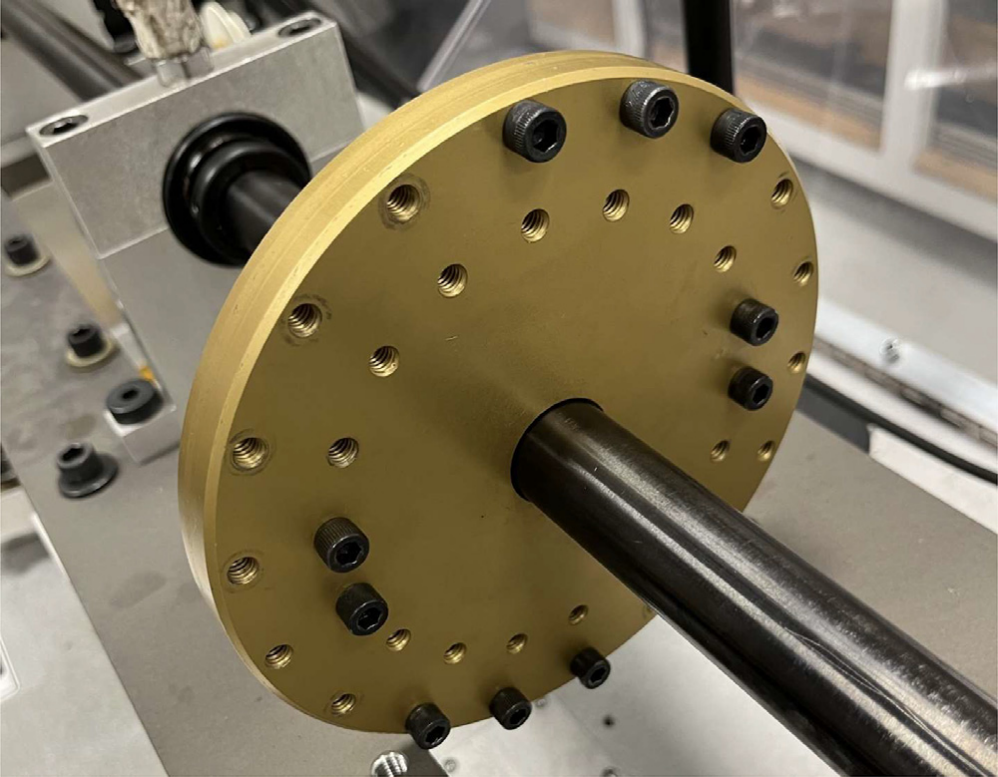
Loaded test rig setup.

Fig. 4 shows the loaded test rig condition, where ten bolts attached symmetrically to a disk mounted on the shaft are added as a load.

**Fig. 5:**
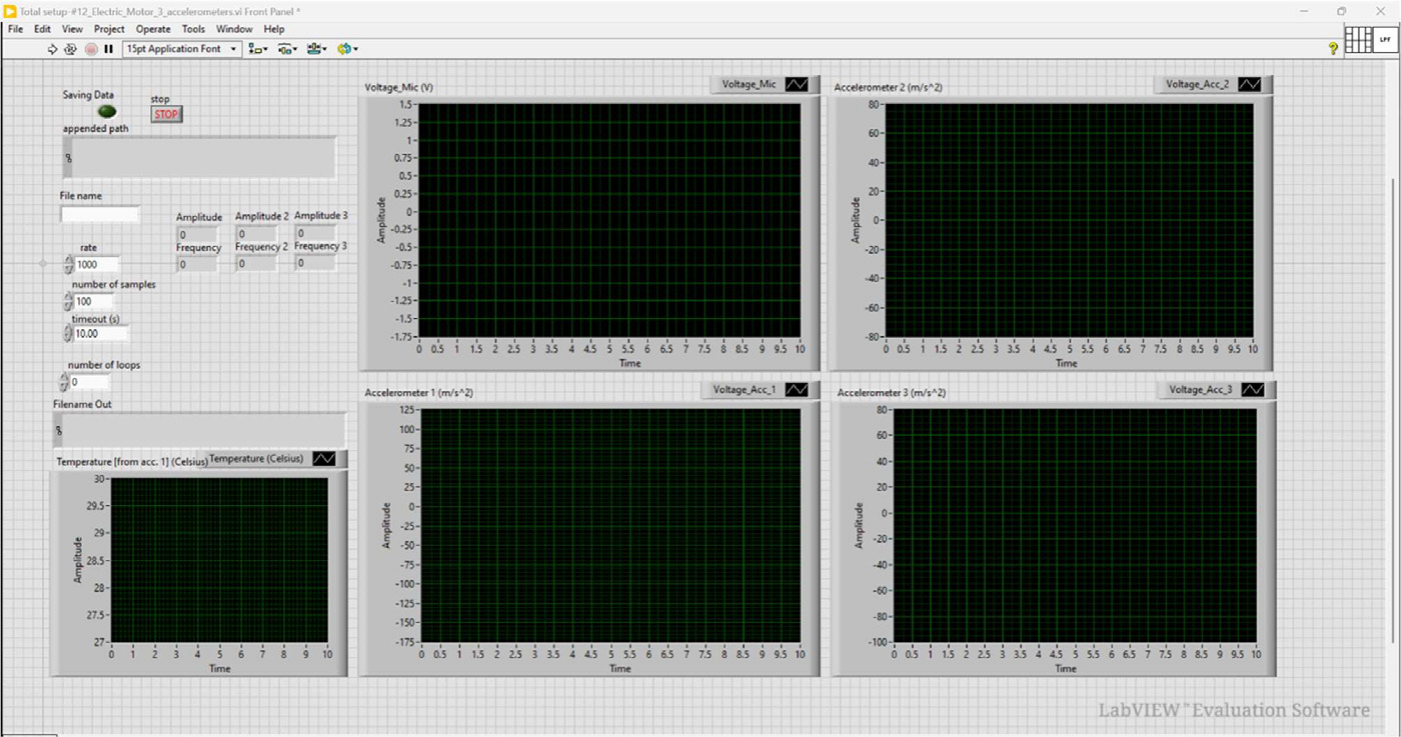
Front panel of the LabVIEW setup.

**Fig. 6:**
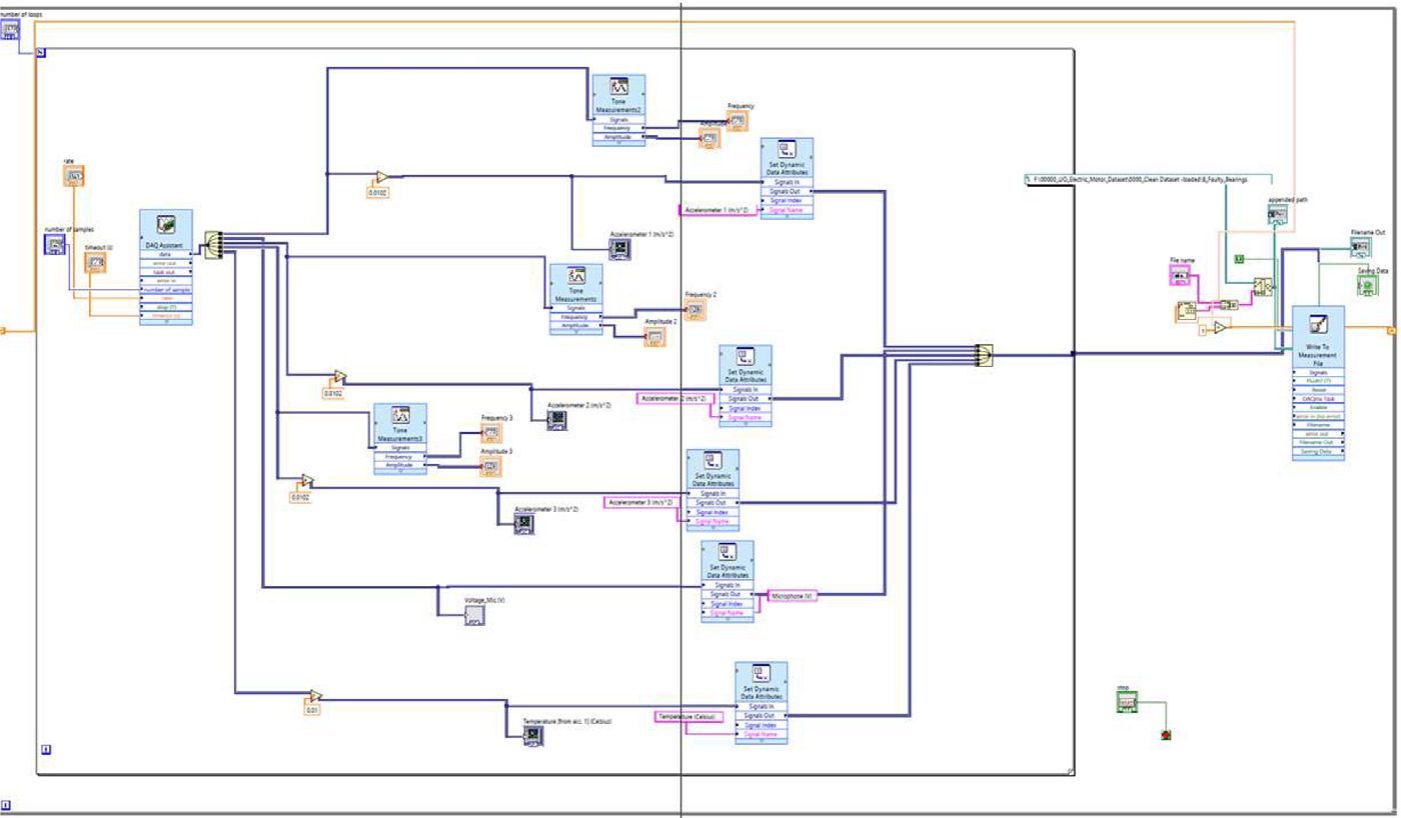
Block diagram of the LabVIEW setup.

The sensors are connected to the computer via a National Instruments USB-6212 data acquisition system. The accelerometers and microphone sensors are connected to a PCB Piezotronics 482C signal conditioner. The accelerometers are used to collect vibration and temperature signals at the drive end and on the shaft of the system, the microphone collects acoustic signals, and the variable speed drive is used to collect the rotational frequency of the motor. LabVIEW's virtual instrumentation software is used to capture the data. Fig. 5 shows the front panel and Fig. 6 shows the block diagram used in this study.

## Limitations

• The dataset provides a limited number of samples for each fault type, as only one motor having each type of fault was available for data collection.
• No filter was applied during signal collection. Therefore, preprocessing of the data may be necessary before it can be used.
• Data collection was conducted in a laboratory environment and may not be representative of industry data.
• Placing the sensor on the motor itself introduces electromagnetic interference caused by the power converter's high switching frequency and pulse width modulation (PWM). The PWM inverter-fed AC motor drive contributes to electrical interference, potentially leading to signal corruption at high frequencies [10]. Consequently, preprocessing of the data acquired from accelerometer 1 on the motor drive end may be necessary before the data can be used.

## CRediT authorship contribution statement

**Mert Sehri:** Conceptualization, Methodology, Validation, Investigation, Data curation, Visualization. **Patrick Dumond:** Writing – review & editing, Supervision.

## Data Availability

University of Ottawa Electric Motor Dataset – Vibration and Acoustic Faults under Constant and Variable Speed Conditions (UOEMD-VAFCVS) (Original data) (Mendeley Data).

## References

[1] Sehri M., Dumond P., Bouchard M. (2023). University of Ottawa constant load and speed rolling-element bearing vibration and acoustic fault signature datasets. Data Br..

[2] M. Sehri and P. Dumond, “University of Ottawa Rolling-element Dataset – Vibration and Acoustic Faults under Constant Load and Speed conditions (UORED-VAFCLS),” vol. 5, 2023, 10.17632/y2px5tg92h.5. 10.1016/j.dib.2023.109327 PMC10331275 37435140

[3] “MAFAULDA: Machinery Fault Database [Online].” (Accessed 15 January 2024). [Online]. Available: https://www02.smt.ufrj.br/~offshore/mfs/page_01.html.

[4] “Apparatus & Procedures | Case School of Engineering | Case Western Reserve University,” Case School Eng. (Accessed 16 February 2023). [Online]. Available: https://engineering.case.edu/bearingdatacenter/apparatus-and-procedures.

[5] Gultekin M.A., Bazzi A. (2023). Review of fault detection and diagnosis techniques for AC motor drives. Energies.

[6] Junior R.F.R., dos S. Areias I.A., Campos M.M., Teixeira C.E., da Silva L.E.B., Gomes G.F. (2022). Fault detection and diagnosis in electric motors using 1d convolutional neural networks with multi-channel vibration signals. Measurement.

[7] Shannon C.E. (1948). A mathematical theory of communication. Bell Syst. Tech. J..

[8] Nyquist H. (1924). Certain Factors Affecting Telegraph Speed. Trans. Am. Inst. Electric. Eng..

[9] M. Sehri and P. Dumond, “University of Ottawa Electric Motor Dataset – Vibration and Acoustic Faults under Constant and Variable Speed Conditions (UOEMD-VAFCVS),” vol. 1, 2023, 10.17632/msxs4vj48g.1. 10.1016/j.dib.2024.110144 PMC11636780 39669982

[10] Jettanasen C. (2012). 2012 15th International Conference on Electrical Machines and Systems (ICEMS).

